# What explains the socioeconomic status gap in activity? Educational differences in determinants of physical activity and screentime

**DOI:** 10.1186/s12889-016-3880-5

**Published:** 2017-02-01

**Authors:** Nelli Hankonen, Matti T. J. Heino, Emilia Kujala, Sini-Tuuli Hynynen, Pilvikki Absetz, Vera Araújo-Soares, Katja Borodulin, Ari Haukkala

**Affiliations:** 1 Department of Social Research, University of Helsinki, Helsinki, Finland; 2 School of Social Sciences and Humanities, University of Tampere, Tampere, Finland; 3 School of Health Sciences, University of Tampere, Tampere, Finland; 4 Institute of Health and Society, Faculty of Medical Sciences, Newcastle University, Newcastle, UK; 5 National Institute for Health and Welfare, Helsinki, Finland

**Keywords:** Socioeconomic status, Adolescents, Physical activity, Screen time, Sedentary behaviour, Theoretical determinants, Theoretical domains framework

## Abstract

**Background:**

Designing evidence-based interventions to address socioeconomic disparities in health and health behaviours requires a better understanding of the specific explanatory mechanisms. We aimed to investigate a comprehensive range of potential theoretical mediators of physical activity (PA) and screen time in different socioeconomic status (SES) groups: a high SES group of high school students, and a low SES group of vocational school students. The COM-B system, including the Theoretical Domains Framework (TDF), was used as a heuristic framework to synthesise different theoretical determinants in this exploratory study.

**Methods:**

Finnish vocational and high school students (*N* = 659) aged 16–19, responded to a survey assessing psychological, social and environmental determinants of activity (PA and screen time). These determinants are mappable into the COM-B domains: capability, opportunity and motivation. The outcome measures were validated self-report measures for PA and screen time. The statistical analyses included a bootstrapping-based mediation procedure.

**Results:**

Regarding PA, there were SES differences in all of the COM-B domains. For example, vocational school students reported using less self-monitoring of PA, weaker injunctive norms to engage in regular PA, and fewer intentions than high school students. Mediation analyses identified potential mediators of the SES-PA relationship in all of three domains: The most important candidates included self-monitoring (CI95 for b: 0.19–0.47), identity (0.04–0.25) and material resources available (0.01–0.16). However, SES was not related to most determinants of screentime, where there were mainly gender differences. Most determinants were similarly related with both behaviours in both SES groups, indicating no major moderation effect of SES on these relationships.

**Conclusions:**

This study revealed that already in the first years of educational differentiation, levels of key PA determinants differ, contributing to socioeconomic differences in PA. The analyses identified the strongest mediators of the SES-PA association, but additional investigation utilising longitudinal and experimental designs are needed. This study demonstrates the usefulness of combining constructs from various theoretical approaches to better understand the role of distinct mechanisms that underpin socioeconomic health behaviour disparities.

## Background

Lack of physical activity (PA) is a major public health problem. Globally, four-fifths of adolescents do not reach recommended levels of PA, i.e. 60–90 minutes a day [1]. Adolescents also engage in unhealthy amounts of sedentary behaviours (SB), especially screen time such as sitting in front of TV, computers and console games, linked to adverse health outcomes independent of PA [2, 3]. Consequently, many national PA guidelines for children and youth additionally include a recommendation of a maximum of two hours of screen time per day, also in Finland [4].

Socioeconomic status (SES) refers to socioeconomic standing in society, measured by educational level, occupation, or income [5]. Educational level is the most frequently used measure of SES in Finland [6], and among adolescents, this means those enrolled in a vocational school (lower SES) versus those in high school (higher SES). High SES is linked with higher levels of PA [7], and physical inactivity is one of the most important behaviours explaining higher mortality in lower SES population [8, 9]. SES differences in PA appear already in youth [10], and worldwide, this difference has increased over the last decade [6, 11].

To address socioeconomic health disparities [12], it is necessary to move beyond description, to increase knowledge on the potential modifiable factors explaining the SES–PA relationship. The known correlates or determinants of PA in youth are such potential mediators.

### Determinants of adolescent physical activity and screen time

Several reviews [13–16] have identified psychosocial and environmental determinants of adolescent PA. As studies tend to refer to different theories and use a multitude of theoretical constructs – although often strongly overlapping – a useful framework for classifying the various determinants is provided by the COM-B model [17]. The COM-B assumes three essential categories of necessary factors for the performance of a specific behaviour, these are: 1) *capability*, an individual’s psychological and physical capacity to engage in a specific behaviour or sets of behaviours, 2) *opportunity*, defined as factors outside an individual that make the behaviour possible or prompt it, and 3) *motivation* to engage in the behaviour [17]. In line with dual process models in psychology, the COM-B distinguishes reflective motivation (e.g., intention) and automatic motivation (e.g., automaticity) as key influences on behaviour, with capability and opportunity also influencing motivation. Other determinants may have differential impacts on motivation, not only on behaviour, thus it is important to investigate indicators of motivation as outcomes.

The COM-B can further be specified with sub-constructs mapped onto the Theoretical Domains Framework (TDF). The TDF was developed based on 128 unique theoretical constructs from 33 different theories, these unique constructs were then aggregated into 14 theoretical domains [18].

Evidence on determinants of PA and of screentime will next be presented, organised under the COM-B domains and the TDF [18, 19] See Appendix 1 for the COM-B categories, Theoretical Domains and the determinants measured in this study.

#### Determinants of physical activity

A subfacet of *capability*, the psychological ability to regulate one’s behaviour meaning ‘anything aimed at managing or changing objectively observed or measured actions’ [18] is important for both initiation and maintenance of behaviour change. A recent review [20] has identified a relationship between behaviour planning and PA among adolescents.

Environmental *opportunity*, i.e. favourable context and sufficient resources are important facilitators of behaviour. Perceived access to PA facilities [13], as well as opportunities for PA in the community [15], and school [16] are positively correlated with PA among adolescents. Social environment plays an important role, too, as parental support for PA and support from significant others, such as siblings and peers, are related with adolescent PA [14, 16]. Support from teachers and coaches, however, do not seem to be as important as that from parents and peers [15].

The *motivation* category contains several determinants underlying these processes directing and energising behaviour. Self-efficacy, i.e. individual’s confidence in his/her ability to be physically active in specific situations, is positively correlated with adolescent PA [13, 16, 21, 22], as is higher perceived behavioural control, i.e. perceived ease of being physically active [13, 21] (correlation among adolescents from a meta-analysis *r* = 0.32)[23]. Beliefs about positive and negative consequences of a behaviour, i.e. outcome expectancies (e.g., [24]), have been linked to changes in adolescent PA, but the evidence is inconclusive [22]. This also applies to evidence for attitudes as determinants of PA [16, 20, 21] (correlation among adolescents from a meta-analysis *r* = 0.36 [23]).

Although intention to engage in PA partially determines adolescent PA [15, 25] (correlation among adolescents from a meta-analysis *r* = 0.46 [23]), there is a well-known gap between intentions and behaviour [26, 27]. Habit strength (automaticity) and identity relevance of a behaviour are factors related to motivation, which have recently gained increasing attention in research on energy-balance behaviours. Already in children, high habit strength is associated with more PA [28]. Those adolescents who identify with the concept of being a physically active person are more likely to engage in regular PA than those who do not [29].

#### Determinants of screen time

Very few high quality studies have investigated determinants of adolescent SBs [16, 20, 30]. Furthermore, screen time consists of different behaviours, which may also have different behavioural determinants. Thus far, TV-viewing has been studied much more than other forms of screen time. Determinants may also vary between target population [31].

##### Opportunity

Among children, parental rules and limitations on screen time have been associated with less screen time, and availability of devices (e.g. TV or a computer in the bedroom) with greater amount of screen time [32, 33]. Various social demographical correlates (e.g. single-parent family as well as low parental income and education) are related with more screen time [16, 30].

##### Motivation

Perceived benefits of SBs (e.g.,enjoyment and the opportunity to unwind) have been linked with resistance to change sedentary habits [34]. Self-efficacy and habit strength also play a role in SBs: youth with higher confidence in their ability to reduce SB are less sedentary [34], while strong TV-viewing habits are related to exceeding the recommended levels of TV-viewing [35].

*Capability* to use the technological equipment required for screentime and TV viewing is easily acquired by all of us, as the technological design of these products relies on cognitive abilities that all humans are capable of developing. Hence, we expect that variables associated with capability for screen time behaviour will not be as impactful.

### Which determinants mediate the influence of SES on activity?

What then could explain the well-documented SES-differences in PA? Socioeconomic differences may be evidenced as different levels in key theoretical determinants, accountable for differing levels of activity (i.e., mediation).

Previous studies are sparse. Among adults, self-efficacy, social support [36, 37], and availability of and access to PA facilities [37–39] are potential candidates. Also, favourable environment for PA may not be equally accessible for those with lower SES [40]. People with higher SES may have greater sense of control over their PA and their health and higher levels of social support (e.g. [41–43]). Higher education may also enhance individuals’ ability to use self-regulatory skills [44].

Socio-structural factors such as SES are often excluded from health behaviour change models [45], a limitation recently acknowledged (e.g., [45, 46]). Several health behaviour theories assume the SES to be a distal influence [24], but this assumption is rarely tested. This study aims at filling the gaps in literature by investigating this assumption explicitly across several potential determinants.

SES differences may arise also from moderation effects. For example, intention had a weaker relationship to prospective behaviour among those with lower SES compared to their high SES counterparts [46], suggesting that those with lower SES may have difficulties in translating healthy intentions into action [46], although findings on this are mixed [47, 48].

#### Aims

**Fig. 1:**
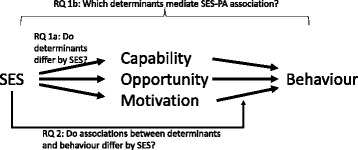
Research questions

The present study will comprehensively investigate theoretical constructs that may explain SES differences in activity behaviours, i.e., moderate-to-vigorous PA (MVPA) and screen time. We make comparisons between a broad range of determinants based on relevant behavioural theories, roviding by the COM-B model and TDF, in a representative sample of vocational and high school students, representing low and high SES youth respectively. First, we investigate whether SES is associated both with the determinants and the behaviours (RQ1a). We also investigate which determinants might mediate the relationship between SES and behaviour (RQ1b). We analyse whether there are differences in determinants by gender, given the expected differences from previous literature both on PA and SB. Secondly, we investigate whether SES moderates the relationships between specific determinants and behaviour (RQ2). See representation of the research questions (RQ) in Fig. 1.

## Methods

Data were collected via an electronic survey among Finnish vocational and high school students during March-April 2013. Data collection took place in schools under teacher’s supervision. Altogether the 13 largest vocational schools from five different areas in Finland were invited to participate in the survey [49]. The largest municipal education and training consortia include the highest number in educational tracks (tourism industry, beauty care, catering, metalwork and machinery), compared to smaller school units, and thus improved the variability of educational tracks in our sample. To enable comparison between the low and high SES groups, six high-schools in the areas of the participating vocational schools were also invited to participate. We aimed for better comparability by inviting the high schools from geographically matching areas. 765 students responded to the questionnaire. 507 students from eight vocational schools (62% of the schools invited agreed to participate) and 152 students from three high schools (50% of the schools invited agreed) fit the age criterion of 16–19 years, and were thus included in the analysis.

### Measures

#### Behavioural determinants

To develop the assessment tool, the determinants were selected utilising previous reviews on the determinants of adolescent PA and sedentary behaviour to map on all relevant domains of the COM-B [17]. Determinants were measured according to specific recommendations [24, 50] and in line with earlier research (e.g., [51]). Table 1 shows the items. Cronbach’s alphas ranged from 0.42 (material resources) to 0.96 (Physical Education (PE) teacher autonomy support and action planning), with most scales at satisfactory levels (see Table 1).

**Table 1:** Measures for determinants of physical activity and screen time

Physical activity			Screen time		
COM-B domain	Measure/example items	Scale (α)	COM-B domain	Measure/example items	Scale (α)
Capability			Capability		
Self-monitoring	Sniehotta et al., 2005	1–7 (.92)			
Action planning	Sniehotta et al., 2005	1–4 (.96)			
Coping planning	Sniehotta et al., 2005	1–4 (.93)			
Knowledge of physical activity recommendations	*How much brisk physical activity do you think is recommended for adolescents aged 15–18*?	1–6	Knowledge of screen time recommendations	*How much screen time - sitting in front of a computer*, *watching TV or playing video games do you think is recommended for adolescents aged 15*–*18*?	1–6
Opportunity			Opportunity		
Access to facilities	‘*There are good paths for cycling and jogging in my environment*.’ ‘*I have plenty of good exercising facilities* (*e.g. sports centres and* –*halls*, *gyms*, *fitness centres*) *in my neighbourhood*.’ ‘*There are good public transport and travel connections to exercising facilities*.’ ‘*I have a lot to do in terms of school*, *hobbies and friends*.’	1–7 (.78)	TV, play console and computer in room	‘*I have a TV*, *play console and/or computer in my room*.’	1–7
Material resources	‘*I have enough money to be physically active*.’ ‘*I don*’*t have the equipment I need for PA*.’	1–7 (.42)			
Injunctive norm	‘*My parents would like me to exercise regularly*’^*a*^	1–7 (.75)	Injunctive norm	‘*My parents would approve of me engaging in screen time more than two hours per day in my free time*’^*a*^	1–7 (.82)
Descriptive norm	‘*Most of my friends exercise regularly*’^*a*^	1–7 (.63)	Descriptive norm	‘*Most of my friends engage in screen time more than two hours per day on their free time*’^*a*^	1–7 (.67)
Parental support	‘*My parents encourage me to be physically active in my free time*.’ ‘*I feel that my parents give me choices*, *options and opportunities to be physically active*.’	1–7 (.88)	Parents restrict screen time No screen time rules at home	‘*My parents restrict my screen time*.’ ‘*There are no rules about the length of screen time in my home*.’	1–7 1–7
PE Autonomy support	Hagger et al., (2009)	1–7 (.96)			
Motivation			Motivation		
Positive outcome expectancy	‘*It would put me in a good mood*’^*a*^	1–7 (.87)	Positive outcome expectancy	‘*I would be informed about what is happening in the world*’^*a*^	1–7 (.83)
Negative outcome expectancy	‘*It would take take too much time from other important things in my life*’^*a*^	1–7 (.74)	Negative outcome expectancy	‘*My neck and upper back muscles would get stiff or sore*’^*a*^	1–7 (.82)
Instrumental attitude	’*Engaging in MVPA three times per week for at least 30 minutes at a time would be useful*… *useless*’^*a*^	1–7 (.92)	Instrumental attitude	‘*Watching TV*, *playing console games and using a computer more than 2 hours per day would be a good* … *bad thing*’^*a*^	1–7 (.93)
Affective attitude	’*Engaging in MVPA three times per week for at least 30 minutes at a time would feel pleasant* … *unpleasant*’^*a*^	1–7 (.55)	Affective attitude	‘*Watching TV*, *playing console games and using a computer more than 2 hours per day would feel pleasant* … *unpleasant*’ ^a^	1–7 (.40)
Intention	‘*I intend to do active sports and*/*or vigorous exercise*, *for at least 30 minutes*, *3 days per week during my free time*, *over the next 4 weeks*’^*a*^	1–7 (.95)	Intention	‘*I intend to watch TV*, *play console games or spend my time on a computer more than two hours a day on weekdays over the next four weeks*/*on weekend over the next 4 weeks*’^*a*^	1–7
PA identity	3 items describing identity from SRHI (Verplanken & Orbell 2003)	1–7 (.66)	ST identity	3 items describing identity from SRHI (Verplanken & Orbell 2003)	1–7 (.67)
Self-efficacy and Perceived behavioural control	‘*If I wanted to*, *I could do active sports and*/*or vigorous exercise three times per week*’^*a*^ ‘*I feel in complete control over whether I will do active sports and*/*or vigorous exercise three times a week*’^*a*^	1–7 (.88)	Self-efficacy and Perceived behavioural control	‘*If I wanted to*, *I could watch TV*, *play console games and spend time on computer more than two hours per day on my free time*’^*a*^ *I feel in complete control over whether I will watch TV*, *play console games or spend time on computer more than two hours per day in my free time*’^*a*^	1–7 (.80)
Habit strength (automaticity)	SRBAI (Gardner et al., 2012)	1–7 (.93)	Habit strength (automaticity)	SRBAI (Gardner et al., 2012)	1–7 (.92)

^a^Measure based on Theory of Planned behavior, Fishbein & Ajzen, 2010 and Francis et al., 2004. The target behaviors were defined in the questionnaire as follows:

PA: ”With physical activity, we mean leisure-time PA that increases your heart rate and makes your breathing get faster. Such PA can be e.g. cycling to school, ball games, running, brisk walking, roller skating, skateboarding, snowboarding, downhill skiing, weight training, aerobics or other group exercise classes, and dancing”

ST: ”With screen time, we mean watching TV, playing console games and spending time on a computer while sitting during leisure time. Spending time on a computer may include e.g. surfing the internet, using social media, chatting with friends via the internet and watching TV and movies and listening to music on the computer. In this survey, time used for homework is not considered screen time”

Other measured variables included self-assessed health and physical condition (both measured on a scale from 1 = very good to 5 = very poor), as well as injuries (yes/no).

#### Behaviours

Self-reported MVPA was assessed with a question: *During the last seven days, on how many days were you physically active so that the activity intensity was moderate or vigorous and you were active at least 30 minutes per one day* (scale 0…7 days). The validity of this question was tested against objectively measured PA in a sub-sample (*n* = 44) of adolescents, using a triaxial accelerometer (Hookie Meter v2.0, Hookie Technologies Ltd, Espoo, Finland). The activity data was registered as raw data at a 100 Hz sample rate in a 2GB internal flash memory. Accelerometers were worn to monitor PA for seven consecutive days. After the week, participants responded to the questionnaire that included the self-reported MVPA question (see above). The correlation coefficient between the Hookie-measured average daily MVPA (approximately above four METs) and the self-reported MVPA was adequate, *r =* .38 (*p* < .02). Self-reported screen time was reported separately for weekday and weekend andassessed with the following questions: *‘How many hours a day during the last 4 weeks have you watched TV on a normal weekday/weekend?’* and *‘How many hours a day during the last 4 weeks have you played console games or used a computer for your free time activities on a normal weekday/weekend?’.* The response alternatives were: ‘*not at all’, ‘0.5 hours per day’, ‘one hour per day’, ‘2 hours per day’, ‘2.5 hours per day’, ‘3 hours per day’, ‘3.5 hours per day’, and ‘4 hours or more per day’.*

#### Statistical analyses

To analyse groups differences (SES) in the assessed theory-driven determinants of PA and screen time, t-tests as well as analyses of variance and covariance were conducted. The interrelationships between the determinants, PA and screen time were analysed using pairwise bivariate correlations. Comparisons of proportions between students estimating the national recommendations correctly and incorrectly were conducted using chi-square tests.

For the parallel multiple mediation analyses, SPSS Statistics 23.0 was used with Hayes’s PROCESS macro (Version 2.15) [52] model 4 (see [53] for full documentation). This OLS regression-based conditional process analysis allows for a maximum of 10 mediators in one test, hence, not all of the mediators were entered in one analysis. Led by the COM-B model, we tested three models, one for each of the COM category, to first identify the most important mediators in each of the categories (Capability, Opportunity, and Motivation). Finally, we tested additional models for sensitivity, in which we included the supported mediators from the first three models and gender as a covariate. Bias corrected bootstrap confidence intervals were created by using 1000 bootstrap samples. This means repeatedly sampling from the original data with replacement and adjusting the interval, based on the skew of the distribution of bootstrap estimates [53]. Calculations for the test of the difference between two independent correlation coefficients [54] were conducted with computer software available at http://quantpsy.org [55]. Model assumptions were tested and fulfilled.

## Results

Participant characteristics are shown in Table 2. Age ranged between 16–19 years (M = 17.8, SD = 0.73). Self-reported health was on average high (*M =* 2.1, *SD. =* .83 for vocational students; *M =* 2.0, *SD =* .68 for high school students). Vocational school students reported poorer physical condition than the high school students (*M =* 2.5, *SD =* .91 for vocational students; *M =* 2.3, *SD =* .88 for high school students). No differences between schools were found in illnesses or injuries limiting PA.

**Table 2:** Descriptives, vocational and high school students

	Vocational school	High School	
	N (%)	N (%)	*p*
Age	507 M = 17.77	152 M = 17.78	0.88
Gender	506	152	0.07
* boy*	232 (45.8)	51 (33.6)	
* girl*	274 (54.2)	101 (66.4)	
Study year	506	152	< .001
* 1st*	228 (45.1)	47 (30.9)	
* 2nd*	167 (33.0)	75 (49.3)	
* 3rd*	107 (21.1)	24 (15.8)	
* 4th*		5 (3.3)	
Self-reported health	473	144	.039
* very good*	103 (21.8)	33 (22.9)	
* good*	243 (51.4)	84 (58.3)	
* average*	104 (22.0)	25 (17.4)	
* poor*	16 (3.4)	2 (1.4)	
* very poor*	7 (1.5)	0.0	
Self-reported physical condition	473	144	.024
* very good*	65 (13.7)	26 (18.1)	
* good*	180 (38.1)	66 (45.8)	
* average*	172 (36.4)	37 (25.7)	
* poor*	48 (10.1)	15 (10.4)	
* very poor*	8 (1.7)	0.0	
Illness or injury limiting PA	474	144	.630
* no*	373 (78.7)	116 (80.6)	
* yes*	101 (21.3)	28 (19.4)	

### Determinants of PA: SES differences

Compared to high school students, vocational school students reported lower weekly frequency of MVPA (Table 3) (Cohen’s *d* = -.33). *Capability.* Self-regulatory behaviours were lower among vocational school students than among high school students, with statistically significant differences in mean levels of self-monitoring *(d* = -.43) and action planning *(d* = -.25), but not in coping planning (*d* = -.06). The current recommendation for PA among 15–18-year-olds was correctly estimated by 14.4%, underestimated by 72.7%, and overestimated by 13.0% of the vocational students in comparison to 26.6%, 62.9% and 10.5% of high school students, respectively (*x* ^*2*^ *= 11.3, df = 2, p =* .004). This means that a larger proportion of high school students had the correct knowledge regarding the national recommendation, and that, compared to high school students, more vocational students estimated the national recommendation to endorse less PA.

**Table 3:** Mean values of PA^a^ and determinants of PA (*N* = 656)

	Vocational school Mean (sd)			High school Mean (sd)						
	Boys (*N* = 231)	Girls (*N* = 273)	Total (*N* = 504)	Boys (*N* = 51)	Girls (*N* = 101)	Total (*N* = 152)	School *p*	School η^2^	Gender *p*	School x Gender interaction *p*
Physical activity^b^	4.0 (1.8)	3.7 (1.6)	3.8 (1.7)	4.5 (1.6)	4.4 (1.8)	4.4 (1.7)	.001	.018	.380	.674
Capability										
*Self*-*monitoring*	4.6 (1.7)	4.8 (1.5)	4.7 (1.6)	5.3 (1.2)	5.3 (1.4)	5.3 (1.4)	.000	.030	.383	.493
*Action planning*	2.8 (.98)	2.7 (.99)	2.7 (.99)	3.2 (.82)	2.9 (.98)	3.0 (.94)	.002	.014	.046	.223
*Coping planning*	2.6 (.94)	2.5 (.91)	2.5 (.92)	3.0 (.83)	2.4 (1.0)	2.6 (1.0)	.081	.005	.000	.005
Opportunity										
*Access to facilities*	5.1 (1.6)	5.1 (1.5)	5.1 (1.5)	5.4 (1.4)	5.2 (1.4)	5.2 (1.4)	.219	.002	.545	.523
*Material resources* (*money*, *equipment*)	5.1 (1.5)	4.8 (1.5)	4.9 (1.5)	5.7 (1.5)	5.2 (1.4)	5.4 (1.4)	.000	.019	.009	.546
*Injunctive norm*	4.3 (1.6)	4.2 (1.5)	4.3 (1.6)	5.1 (1.3)	4.8 (1.4)	4.9 (1.4)	.000	.034	.163	.450
*Descriptive norm*	4.4 (1.3)	4.3 (1.1)	4.4 (1.3)	4.7 (1.2)	4.6 (1.4)	4.6 (1.3)	.016	.009	.558	1.0
* Parental support*	5.0 (1.7)	4.9 (1.7)	5.0 (1.7)	5.4 (1.3)	5.3 (1.7)	5.3 (1.6)	.014	.009	.422	.978
*PE Autonomy support*	5.1 (1.5)	5.1 (1.5)	5.1 (1.5)	5.1 (1.5)	5.1 (1.4)	5.1 (1.4)	.996	.000	.745	.966
Motivation										
*Positive OE*	5.0 (1.4)	5.4 (1.4)	5.2 (1.2)	5.3 (1.2)	5.6 (1.1)	5.5 (1.2)	.077	.005	.001	.560
*Negative OE*	3.4 (1.3)	3.3 (1.3)	3.3 (1.3)	2.9 (1.2)	2.9 (1.2)	2.9 (1.2)	.000	.020	.322	.591
*Instrumental attitude*	6.1 (1.3)	6.6 (.86)	6.3 (1.1)	6.4 (.86)	6.7 (.57)	6.6 (.69)	.010	.010	.000	.361
*Affective attitude*	5.0 (1.4)	5.4 (1.4)	5.2 (1.4)	5.4 (1.2)	5.9 (1.4)	5.7 (1.3)	.002	.015	.002	.532
*Intention*	5.0 (1.9)	5.4 (1.6)	5.2 (1.8)	6.0 (1.2)	5.8 (1.7)	5.9 (1.6)	.000	.029	.579	.085
*PA identity*	4.7 (1.4)	4.5 (1.3)	4.6 (1.3)	5.0 (1.5)	5.0 (1.6)	5.0 (1.5)	.001	.017	.390	.432
*SE & PBC*	5.8 (1.5)	6.0 (1.4)	5.9 (1.4)	6.5 (.71)	6.2 (1.3)	6.3 (1.1)	.001	.017	.744	.039
*Automaticity*	4.5 (1.6)	4.3 (1.6)	4.4 (1.6)	4.8 (1.5)	4.5 (1.8)	4.6 (1.7)	.150	.003	.106	.830

^a^ *PA* physical activity, *OE* outcome expectancy, *PE* physical education, *SE* self-efficacy, *PBC* perceived behavioural control

^b^Days per week with > 30 min MVPA

#### Opportunity

Vocational students reported less material resources (e.g. money, equipment) for PA than high school students *(d* = -.30), but differences were not detected regarding access to PA facilities *(d* = -.10). The social environment was less supportive of PA among vocational students: subjective norms, both injunctive *(d* = -.43) and descriptive *(d = -.*23), as well as parental support for PA *(d = -.*23) were lower than among high school students. No differences were detected in the amount of autonomy support the groups reported getting from their current PE teacher (.00 *< d < .01*).

#### Motivation

Vocational students had more negative outcome expectancies *(d* = .35), and less favourable instrumental *(d* = -.27) and affective attitudes *(d = -.*35) towards PA. Their intentions to be physically active were lower as were their ratings of their PA identity (*d* = -.39), self-efficacy and perceived behavioural control (*d* = -.28). No significant SES differences were found in positive outcome expectancies (*d* = -.20) and automaticity *(d = -.*11).

#### Gender differences and interactions

Boys reported having more material resources for PA than girls did (*p* = .009), but girls displayed more positive outcome expectancies and attitudes than boys (*p* < .01). Self-efficacy was highest among high school boys, and lowest among vocational school boys (*p =* .039).

#### Determinants of screen time: SES differences

Compared to high school students, vocational students reported more leisure screen time on weekdays but not on weekend (Table 4)*.*

**Table 4:** Mean values of screen time and determinants of screen time

	Vocational school Mean (sd)			High school Mean (sd)						
	Boys (*N* = 201)	Girls (*N* = 256)	Total (*N* = 457)	Boys (*N* = 48)	Girls (*N* = 96)	Total (*N* = 144)	School *p*	School η^2^	Gender *p*	School x Gender interaction *p*
Screen time										
* Weekday*	3.5 (2.2)	2.8 (1.8)	3.1 (2.0)	3.2 (1.6)	2.3 (1.6)	2.6 (1.6)	.039	.007	.000	.708
* Weekend*	3.7 (2.2)	3.3 (2.0)	3.5 (2.1)	3.8 (1.7)	3.4 (1.8)	3.5 (1.8)	.707	.000	.040	.907
Opportunity										
* Injunctive norm*	4.7 (1.7)	4.5 (1.8)	4.6 (1.7)	4.9 (1.6)	4.6 (1.8)	4.7 (1.8)	.293	.002	.189	.754
* Descriptive norm*	4.4 (1.4)	4.2 (1.7)	4.3 (1.6)	4.6 (1.4)	4.2 (1.7)	4.4 (1.6)	.473	.001	.056	.551
* Parents restrict screen time*	3.0 (1.7)	2.5 (1.8)	2.7 (1.8)	3.3 (1.7)	2.7 (1.9)	2.9 (1.8)	.118	.004	.003	.649
*No screen time rules at home*	4.7 (2.0)	4.9 (2.2)	4.8 (2.1)	4.6 (1.9)	4.9 (2.1)	4.8 (2.0)	.888	.000	.205	.620
*TV*, *play console and computer in room*	4.9 (2.1)	4.5 (2.3)	4.7 (2.2)	5.2 (2.1)	3.9 (2.3)	4.4 (2.3)	.599	.000	.000	.044
Motivation										
* Positive OE* ^*a*^	4.7 (1.4)	4.7 (1.4)	4.7 (1.4)	5.3 (1.2)	4.9 (1.2)	5.1 (1.2)	.003	.014	.087	.136
* Negative OE*	4.3 (1.4)	4.9 (1.5)	4.6 (1.5)	4.1 (1.5)	5.0 (1.4)	4.7 (1.5)	.976	.000	.000	.400
* Instrumental attitude*	3.5 (1.6)	2.9 (1.6)	3.2 (1.6)	3.3 (1.4)	2.6 (1.5)	2.9 (1.5)	.123	.004	.000	.820
* Affective attitude*	4.0 (1.3)	3.4 (1.3)	3.7 (1.3)	4.0 (1.4)	3.3 (1.5)	3.6 (1.5)	.964	.000	.000	.908
* Intention*	4.5 (1.9)	3.9 (2.0)	4.1 (1.9)	4.6 (1.9)	4.0 (2.2)	4.2 (2.1)	.523	.001	.003	.954
* ST identity*	4.3 (1.3)	4.2 (1.3)	4.2 (1.3)	4.6 (1.1)	4.3 (1.6)	4.4 (1.5)	.103	.004	.150	.369
* SE & PBC*	5.4 (1.6)	5.6 (1.6)	5.5 (1.6)	5.8 (1.5)	5.7 (1.7)	5.8 (1.6)	.122	.004	.766	.479
* Automaticity*	4.1 (1.5)	4.4 (1.7)	4.2 (1.6)	4.7 (1.2)	4.3 (1.9)	4.4 (1.7)	.081	.005	.696	.028

^a^OE = outcome expectancy, ST = screen time, SE = self-efficacy, PBC = perceived behavioural control

##### Capability

Altogether 36.6% of the vocational school and 47.2% of high school students correctly estimated the screen time recommendation. It was under-estimated by 56.1% of the vocational and by 45.8% of the high school students (*x* ^*2*^ *=* 5.2, *df = 2, p* = .075).

##### Opportunity

There were no statistically significant differences between vocational school and high school students in the variables used to measure opportunities to engage in screen time.

##### Motivation

The only difference in motivational correlates of screen time between vocational school and high school students was found regarding outcome expectancies: high school students had more positive outcome expectancies towards screen time than vocational school students *(d* = -.25). No significant SES differences were detected in screen time automaticity *(d* = -.12).

#### Gender differences and interactions

In both vocational and high schools, boys reported more screen time than girls both on weekdays and weekend, and also better material resources and higher motivation for screen time than girls did (see Table 4). The pattern of results in determinants was in line with this finding: compared to boys, girls reported lower availability of screens, more negative outcome expectancies, as well as less positive instrumental and affective attitudes (all *p* < .001). High school boys reported stronger screen time automaticity than vocational school boys (*p* = .028).

#### Mediation analyses

We constructed three models to investigate how the effect of SES might be mediated on PA, one for each of the Capability, Opportunity, and Motivation dimensions (see Appendix 2 for the individual path coefficients). For screen time, no mediation analyses were carried out because no SES differences were detected.

For the capability-model, self-monitoring accounted for most of the effect of SES on PA. The total indirect effect of SES on PA via self-monitoring was *b* = .33, with a 95% bias corrected and accelerated confidence interval (BCa CI) of [.19, .51]. Direct effect of SES on PA was .23, 95% BCa CI [−.04, .50]. Thus, we cannot rule out self-monitoring as a mediator.

For the opportunity-model, four mediators were not excluded: material resources (*b* = .07, 95% Bca CI [.02, .16]), injunctive norms (*b* = .06, 95% BCa CI [.00, .15]), descriptive norms (*b* = .04, 95% BCa CI [.00, .11] and parental support (*b* = .03, 95% BCa CI [.00, .09]). Direct effect of SES on PA in this model was .38, 95% BCa CI [.07, .69].

For the motivation-model, intention (*b* = .20, 95% BCa CI [.11, .33]) and PA identity (*b* = .13, 95% BCa CI [.05, .25]) were not excluded as mediators. Direct effect of SES on PA was .30, 95% BCa CI [.02, .57].

In summary, self-monitoring (Capability), material resources, injunctive and descriptive norms, parental support (Opportunity), as well as intention and identity (Motivation) were found to potentially mediate the relationship between SES and physical activity.

An additional cross-dimensional sensitivity analysis was carried out. In this model, we included the mediators which were supported by the individual-domain mediation analyses presented above. In this model, only Self monitoring (*b* = .17, 95% BCa CI [.08, .27]), Intention (*b* = .12, 95% BCa CI [.03, .23]) and PA identity (*b* = .11, 95% BCa CI [.04, .21]) had CIs that excluded zero.

Adding gender as a covariate did not affect results in any of the models. All Variance Inflation Factors (VIFs) were under 4, not indicating strong multicollinearity problems.

#### Differences in the associations

As a second research question, we investigated whether the strength of correlations varied across vocational and high scool students. For PA, all of the measured determinants except the knowledge of PA recommendation correlated with weekly PA frequency (see Table 5). The highest correlations to PA were by self-monitoring *(r =* .52*, p < .*01), intention *(r* = .49, *p < .*001) and PA identity *(r =* .48*, p <.* 001). On the whole, the correlations were similar among high-school and vocational school students, except for one variable: The self-reported environment and access to PA facilities correlated significantly with PA among vocational school students *(r =* .20*, p <* .01) but not among high school students *(r = -.*02, *p =* .776).

Weekday and weekend screen time were highly inter-correlated among both SES groups (*r* = .72*, p* < .01) (Table 6). The highest correlations to screen time were by intention (weekday *r =* .41, *<.01*; weekend *r* = .46*, p < .*01), automaticity (weekday *r = .*30*, p* < .01; weekend *r =* .35*, p <* .01), and instrumental attitude (weekday *r* = .39, *p* < .01; weekend *r* = .32, *p* < .01). The correlation coefficients among vocational and high school students were again largely similar. Positive outcome expectancy had a significantly larger correlation with weekend screen time among high school students *(r =* .42*, p* < .01) than vocational school students *(r =* .24*, p <* .01).

**Table 5:** Pairwise correlations between PA, intention, automaticity and other determinants of PA

	PA	Intention	Automaticity
PA	1.0		
Intention	.49**	1.0	
Automaticity	.43**	.55**	1.0
*Capability*			
Knowledge of recommendations	.04	−.05	−.11**
Action planning	.42**	.56**	.54**
Coping planning	.34**	.41**	.52**
Self-monitoring	.52**	.70**	.61**
*Opportunity*			
Access to facilities	.16**^a^	.36**	.28**
Material resources	.25**	.31**	.33**
Injunctive norm	.25**	.36**	.35**
Descriptive norm	.26**	.38**	.46**
Parental support	.26**	.40**	.42**
PE autonomy support	.20**	.38**	.34**
*Motivation*			
Positive outcome expectancy	.24**	.44**	.42**
Negative outcome expectancy	−.21**	−.40**	−.29**
Instrumental attitude	.17**	.41**	.24**
Affective attitude	.34**	.54**	.46**
PA identity	.48**	.58**	.64**
Self-efficacy & perceived behavioural control	.30**	.55**	.43**

**Correlation is significant at the 0.01 level (2-tailed). *Correlation is significant at the 0.05 level (2-tailed).

^a^the test of the difference between two independent correlation coefficients for vocational school- and high school is significant (*p* < .05).

## Discussion

We explored socioeconomic differences in a wide range of determinants of adolescent physical activity and screen time, using the Capability-Opportunity-Motivation-Behaviour model as a heuristic framework (COM-B; [17]), and investigated potential mediators for PA. We also examined whether there are SES differences in the strengths of the associations between these determinants and the respective behaviours. Regarding PA, SES differences were found in all of the COM-B domains, in 13 determinants out of 17 measured. Vocational students reported, for example, less self-monitoring, lower injunctive norms and intentions than high school students. Regarding screen time, however, there were only two modestly statistically significant differences attributable to SES; there were substantially more gender differences in the levels of screen time determinants, than SES differences. The mediation analysis pointed to importance of self-monitoring in explaining the link between SES and PA. Also resources, norms as well as intention and identity emerged as statistically significant mediators of the effect. In the final mediation analyses, PA identity, intention and self-monitoring remained significant mediators of the SES-PA-relationship. We found no evidence of significant moderating effects, which implies that the determinants are equally relevant in both SES groups.

Previous research suggest that SES differences in adult PA are explained by availability of and access to PA facilities [37–39], social support and self-efficacy [36, 37]. Our results are partially in line with these. Regarding *capability*, high SES adolescents engaged in more self-monitoring and action planningthe boys also in coping planningof their PA, with the findings supporting earlier suggestions of a link between education and self-regulation [44]. Self-monitoring has earlier been shown to be a key behaviour change technique characterising effective interventions to change PA (e.g. [56], and ours is among the first studies to demonstrate its role in explaining socioeoconomic gap in activity. Those with lower SES may typically experience higher levels of stress from various sources, mainly related to economic factors. This might lead to a more short-term approach to life (time perspective, e.g. [57]) or limitation in cognitive function [58]. These aspects were not directly measured here, but SES differences in self-regulatory constructs such as planning and self-monitoring may also reflect such socioeconomic discrepancies.

High SES adolescents, who had better *opportunities* for PA, i.e.greater material resources and supportive social environment, also exercised more, again in line with previous research [36]. It has been suggested that individuals with a low socio-economic background have poorer access to and availability of PA facilities, reducing their PA [38, 39]. The mediation analyses pointed to four mediators of the SES-PA effect in the Opportunity category: material resources for PA, descriptive and injunctive norms, and parental support. Such material and cultural environmental barriers have emerged also in the literature.

Regarding *motivation*, self-efficacy seems to play an important role in explaining SES differences in adolescent PA, as among adults [36, 37]. However, the mediation analyses did not point to self-efficacy as a key mediator. In the domain of motivation, intention (which is, on the other hand, hypothesised to be influenced by self-efficacy in many theories, e.g. [24]) and identity were found to be potential mediators of on PA.

This study revealed that already in the first years of educational differentiation, levels of key PA determinants differ (as adolescents’ SES was based on their own educational path rather than defined based on their parents’ SES). Interestingly, no SES differences were detected in screen time determinants, contrary to some evidence [59]. Screen time may be better explained by gender than SES – boys have been shown to engage in more screen time than girls [16].

Despite some evidence of SES moderating e.g. intention-behaviour relationship [46], these results were in line with the more recent meta-analysis [48], suggesting that PA intentions lead to behaviour similarly regardless of educational background. Also, another Finnish study [42] has showed that self-efficacy, action planning, coping planning, and social support had similar effects on behaviour among both high and low educated adults. Only three correlations differed between the SES groups, suggesting that intervening on PA and screen time determinants may have similar effects irrespective of SES. Our study also showed an absence of relationship between correct knowledge of national PA recommendations and PA behaviour.

This study has several implications for practice and policy. Considering the wide-echoed political concern about socioeconomic inequalities in health, our study, if replicated in more robust designs, may inform policy to reduce the SES gap in PA and consequently of future health outcomes. This may include acknowledging the heightened needs vocational school students have regarding, especially, self-regulatory skills to plan and monitor their PA. Secondly, lower SES adolescents may currently not be provided with as much social and material support to be physically active as higher SES youth. Lack of environmental resources may be reflected in psychological determinants such as intention and identity. Experimental designs could thus further test whether providing more opportunities and prompting social acceptance for PA, as well as financial support for PA equipment is effective in SES-targeted interventions.

One explanatory mechanism for the lower PA in youth with lower education were the lower ratings of PA-related identity. This is in line with, for example, self-determination theory and evidence on the key role of integrated and identified motivational regulations for PA [60]. It should be noted that PA behaviour may also be influencing the motivation mediators, in a cyclical fashion. Thus, it is unlikely that simply by targeting for example identity and self-monitoring, the socioeconomic difference in PA would disappear – rather, it is likely that factors such as cultural conceptions and childhood PA behaviour manifest themselves in perceived self-identity. However, it is beyond the scope of the present study examine how long-term, societal level processes are causing SES differences in determinants of PA and PA itself.

From the standpoint of developing an intervention, the investigation at hand represents an important first step toward understanding the target behaviour (e.g., PA) and the behavioural determinants in the target population (adolescents) [61]. Table 5 demonstrates a multitude of correlates of both PA behaviour as well as motivation (intention and automaticity). Creating an intervention requires additionally identifying the best intervention methods or techniques to influence these determinants (see e.g [62]). For this purpose, experimental studies and meta-analyses of interventions provide further evidence (e.g. [63]).

**Table 6:** Pairwise correlations between screen time, intention, automaticity and other determinants of screen time

	Weekday screen time	Weekend screen time	Intention	Automaticity
Weekday screen time	1.0			
Weekend screen time	.72**	1.0		
Intention	.41**	.46**	1.0	
Automaticity	.30**	.35**	.50**	1.0
*Capability*				
Knowledge of recommendations	.09*^a^	.13**	.13**	.04
*Opportunity*				
Parents restrict screen time	−.04	−.05	−.12**	−.04
No screen time rules at home	−.01	.01	.11**	.13**
TV, play console and/or computer in room	.14**	.13**	.14**	.15**
Injunctive norm	.20**	.28**	.42**	.44**
Descriptive norm	.24**	.25**	.42**	.42**
*Motivation*				
Positive outcome expectancy	.21**	.27**^a^	.47**	.40**
Negative outcome expectancy	−.22**	−.16**	−.24**	−.04
Instrumental attitude	.39**	.32**	.45**	.24**
Affective attitude	.31**	.32**	.49**	.30**
Screen time identity	.26**	.32**	.49**	.51**
Self-efficacy & perceived behavioural control	.09*	.20**	.28**	.32**

**Correlation is significant at the 0.01 level (2-tailed). *Correlation is significant at the 0.05 level (2-tailed).

^a^the test of the difference between two independent correlation coefficients is significant (*p* < .05).

The differences between correlation coefficients are calculated with Preacher, K. J. (2002, May). Calculation for the test of the difference between two independent correlation coefficients [Computer software]. Available from http://quantpsy.org

The current study informed the development of an intervention for vocational school students [64]. In such development work, the levels of the most important PA determinants among the high-school students could be used as “benchmarks” to identify potential intermediate targets that are relevant, yet potentially changeable, also among the vocational school youth. Yet, such benchmarking should not override a key intervention design principle of understanding the behaviour, needs and resources of the target group in context.

Limitations includes the use of self-report measures of behaviour, skills and environment, thus subject to bias. However, self-report measures are a feasible and a cost-effective way to gather data in a large group, and the data from the subsample assessed with the concomitant use of accelerometers showed that the correlation between the self-reported measure of PA and accelerometer was moderate. Secondly, with multiple tests, the possibility of chance findings and Type 1 errors exist [65]. Thus, although our findings generally are in line with both theory and earlier evidence, these results should be interpreted with caution. Only 62% of the vocational schools and 50% of the high schools invited to participate in the survey finally participated. It may be that the teachers advocating and already promoting a physically active lifestyle more easily offered their students the opportunity to respond to the survey as participation was voluntary and took place during school hours. This may have affected the results in a way that the differences between the two SES groups were slightly smaller than in national surveys [66]. Fourth, the mediation tests were not optimal in that they were conducted within each of the categories. However, models containing all of the variables in the same multivariate model would not have been feasible. Also, the tests were guided by the theoretical domains framework (TDF) and the COM-B: these are among the first tests that examine a wide variety of theoretical predictors in an integrated way, a trend toward which psychological science is currently progressing. We believe that such exploratory, comprehensive studies are important in helping advance theorising about socioeconomic health disparities. A further limitation resides in the cross-sectional and observational design (see also [67, 68]). Given this, no conclusions regarding the causal relationship between the measured theory-based constructs and behaviours can be made. Correlational findings are consistent with the identified variables serving as mediators of the causal relationships between SES and physical activity. This warrants additional investigation utilising longitudinal and experimental designs. Finally, these results may not be generalisable to other populations, e.g. age groups, and further studies are warranted in various subgroups, countries and cultures.

The strengths of this study include coverage of a wide range of determinants for two distinct forms of activity behaviours, enabling a comprehensive investigation of potential explanations for SES differences. Previous investigations have focused on a limited set of determinants, and thus used a narrow conceptualization of the range of influences on behaviour. We also examined a wide range of determinants of screen time, an understudied topic [20]. The sample size was large enough to detect statistically significant differences in the determinants that are also meaningful in practice. However, effect sizes were not very large, indicating to a wide heterogeneity within both groups.

Similar studies are emerging to build the evidence base for designing interventions sensitive to PA and SB determinants critical to low-SES individuals (e.g., [69]), but more are needed. Future studies should investigate SES differences in determinants in different ages to understand whether and how their role changes over the life course. We also recommend including measures other than self-report, e.g., computerised measurements of implicit attitudes and motivations. Preliminary evidence indicates that interventions may induce differential effects in PA motivation for low and high SES youth [70], hence, we encourage such intervention process evaluations sensitive to SES, to identify mechanisms responsible for possibly different outcomes for low and high SES participants.

## Conclusions

SES differences emerged in the domains of capability, opportunity and motivation for PA, but screen time behaviour determinants are better explained by gender than SES. To our knowledge, this was the first study to systematically examine SES differences in a range of known determinants of adolescent PA and screen time as well as on the behaviours. Investigating the SES differences in not only behaviours but also in behavioural determinants makes a crucial contribution in the efforts to better understand the origins of social inequalities in health. Such analysis enables identifying and targeting the most important determinants in interventions to reduce health inequalities.

## Appendix 1

ᅟ

**Table 7:** Theoretical determinants of this study classified by COM-B categories and theoretical domains.

COM-B domain	Theoretical domain	Physical activity determinant measure	Screen time determinant measure
Physical CAPABILITY	Physical skill	-	-
Psychological CAPABILITY	Knowledge Cognitive and int.skills Memory, attention, decision processes Behavioural Regulation	Knowledge of recommendation - - Action & coping planning, self-monitoring	Knowledge of recommendation - - -
Automatic MOTIVATION	Reinforcement Emotion	- Affective attitude Automaticity	- Affective attitude Automaticity
Reflective MOTIVATION	Identity Beliefs about capabilities Optimism Intention Goals Beliefs about consequences	Identity Self-efficacy, PBC - Intention - Outcome expectations, instrumental att.	Identity Self-efficacy, PBC - Intention - Outcome expectations, instrumental att.
Social OPPORTUNITY	Social influences	Injunctive & descriptive norms (peers, parents) Parental support PE teacher autonomy support	Injunctive & descriptive norms (peers, parents) Parental restriction & rules PE teacher autonomy support
Physical OPPORTUNITY	Environmental context and resources	Access to PA facilities Material resources	TV, play console and computer in own room

Note. PBC = Perceived Behavioural Control, PE = Physical Education

## Appendix 2

ᅟ

**Table 8:** Individual path coefficients for mediation analysis.

	Total effect on PA		Variable - > PA		SES - > Variable	
	β	CI95	β	CI95	β	CI95
Model 1: Capability (R^2 = 0.29)						
SES	0.59	[0.28, 0.90]	0.23	[−0.04, 0.50]		
Action planning			0.12	[−0.10, 0.34]	0.22	[0.04, 0.40]
Coping planning			0.10	[−0.09, 0.30]	0.05	[−0.12, 0.22]
Self-monitoring			0.48	[0.38, 0.58]	0.68	[0.40, 0.97]
Model 2: Opportunity (R^2 = 0.13)						
SES	0.58	[0.26, 0.89]	0.38	[0.07, 0.69]		
Access to facilities			0.00	[−0.10, 0.09]	0.14	[−0.14, 0.42]
Material resources			0.15	[0.05, 0.24]	0.48	[0.19, 0.76]
Injunctive norm			0.10	[0.00, 0.20]	0.64	[0.36, 0.92]
Descriptive norm			0.14	[0.01, 0.27]	0.26	[0.02, 0.49]
Parental support			0.08	[−0.02, 0.18]	0.33	[0.02, 0.64]
PE Autonomy support			0.11	[0.02, 0.20]	0.02	[−0.27, 0.30]
Model 3: Motivation (R^2 = 0.32)						
SES	0.60	[0.29, 0.92]	0.30	[0.02, 0.57]		
Affective attitude			0.03	[−0.09, 0.14]	0.48	[0.23, 0.74]
Instrumental attitude			−0.07	[−0.21, 0.07]	0.26	[0.07, 0.44]
Habit			0.15	[0.05, 0.25]	0.18	[−0.12, 0.48]
Intention			0.30	[0.21, 0.39]	0.67	[0.35, 0.99]
Negative OE			0.07	[−0.03, 0.17]	−0.45	[−0.69,−0.22]
PA identity			0.29	[0.17, 0.40]	0.46	[0.19, 0.72]
Positive OE			−0.04	[−0.14, 0.06]	0.26	[0.01, 0.51]
SE & PBC			−0.01	[−0.12, 0.09]	0.39	[0.14, 0.64]

## Abbreviations

PA: Physical activity
SES: Socioeconomic status
TDF: Theoretical Domains Framework
